# Shaping the next era of stem cell science: a vision for stem cells journal and its global community

**DOI:** 10.1093/stmcls/sxaf080

**Published:** 2025-12-10

**Authors:** Majlinda Lako

**Affiliations:** Biosciences Institute, University of Newcastle—Institute of Human Genetics, Central Parkway International Centre for Life United Kingdom

As we approach the 43rd anniversary of the founding of *Stem Cells*, the first journal dedicated to this transformative field, established in 1983, I am deeply honored to assume the role of Editor-in-Chief. Having served for 15 years as an Associate Editor, I have witnessed firsthand the remarkable and rapid progress in pluripotent stem cell biology, gene editing technologies, adult stem cell therapies, and regenerative medicine. Over the past few decades, the field has evolved from foundational discoveries of stem cell identity and function to cutting-edge translational research, promising revolutionary therapies. It is with great excitement and responsibility that I take on this leadership role to continue advancing high-caliber scholarship in stem cell science and its applications worldwide.

My vision for *Stem Cells* is to elevate it as a premier, open-access journal known for publishing rigorous, high-impact research that advances stem cell science conceptually and methodologically. While the field has seen significant progress and many foundational advances, the journal will now emphasize rapid, transparent editorial decisions, and prioritize studies bridging basic and translational research, especially in emerging areas such as regenerative medicine, organoids, and gene therapy. I aim for *Stem Cells* to be the field’s authoritative voice, fostering ethical discourse, policy commentary, and global collaboration. By embracing technical innovations, confirmatory studies, and promoting reproducibility, the journal will improve transparency. Engagement with the community will be enhanced through webinars, podcasts, and special issues, ensuring continuous refinement based on feedback. Citation trends will be monitored to highlight influential work, and initiatives like community review will ensure efficiency and openness. Maintaining a diverse and international editorial board will help attract top-tier submissions and position *Stem Cells* at the forefront of innovation and excellence in stem cell research.

Supporting early-career researchers (ECRs) is essential to the growth and diversity of the stem cell field, and *Stem Cells* is committed to providing platforms that empower these emerging scientists. Through initiatives like mentoring programs, ECR section editors, and recognition of outstanding contributions, the journal aims to provide ECRs with opportunities to publish, engage in peer review, and gain editorial experience. This commitment is grounded in fostering an inclusive, transparent publishing environment where open access and data sharing help maximize the impact of ECR research on both science and society. By nurturing the next generation through community engagement, mentorship, and visibility, *Stem Cells* will help shape future leaders and uphold scientific excellence and innovation throughout the field. Promoting diversity in science is essential for driving innovation, fostering creativity, and ensuring that research addresses the needs of a global community. I am committed to building inclusive research teams and supporting scientists and reviewers from a wide range of backgrounds, experiences, and perspectives.

As we approach the 20th anniversary of induced pluripotent stem cell (iPSC) biology, it is a fascinating time for the field. Since Shinya Yamanaka’s pioneering discovery in 2006 that mature somatic cells could be reprogrammed into pluripotent stem cells using defined transcription factors, the field has witnessed revolutionary advances. iPSCs have transformed basic research by enabling disease modeling, drug discovery, and personalized medicine, and they have opened unprecedented avenues for regenerative therapies, including groundbreaking clinical trials for conditions such as macular degeneration and Parkinson’s Disease. This anniversary not only celebrates two decades of remarkable scientific progress but also highlights the immense potential of iPSCs to continue reshaping biomedical science and patient care in the years ahead. To celebrate these exciting milestones, we will be organizing a special issue dedicated to the 20th anniversary of iPSC biology. This issue will showcase landmark achievements and highlight novel advances that continue to propel the field forward, offering a comprehensive perspective on the transformative impact of iPSCs across basic science and clinical applications.

I would like to express my sincere appreciation for the outstanding leadership and dedication of Dr Jan Nolta, the former Editor-in-Chief of *Stem Cells*. With over three decades of experience in human stem cells, gene therapy, and clinical trial development, Jan has significantly advanced the field through her prolific research and mentorship. Her tireless commitment to scientific excellence and fostering a vibrant research community has left a lasting impact on the journal and the broader stem cell field. We owe her an outstanding debt of gratitude for guiding STEM CELLS to its esteemed position in the scientific world.

Teamwork lies at the heart of progress in stem cell science. In this new role, I am privileged to be joined by Associate Editors Ruby Shalom-Feuerstein (Technion Israel Institute of Technology, Israel), Ralf Jauch (The University of Hong Kong, China), and Robert Zweigerdt (Hannover Medical School, Germany). Ruby brings a distinguished background in epithelial stem cell biology and tissue regeneration, with impactful contributions to understanding the function of skin and corneal stem cells. Ralf contributes deep expertise in transcriptional regulation, chromatin architecture, and the molecular control of pluripotency and cellular identity, illuminating how regulatory networks guide cellular reprogramming and fate decisions of stem cells. Robert adds exceptional strengths in pluripotent stem cell bioprocessing for regenerative medicine and advanced organoid systems that uncover the mechanisms of cellular differentiation and tissue patterning during early human development in vitro. Their complementary expertise and shared dedication to scientific excellence make them invaluable partners in leading *Stem Cells* forward.

Together, we aim to foster innovation, uphold rigorous peer review, and promote an inclusive global community of stem cell researchers. We warmly invite investigators worldwide to submit their most compelling and visionary work to *Stem Cells* and join us in shaping the next frontiers of stem cell and regenerative science.

## Conflicts of interest

None declared.

